# Static FET PET radiomics for the differentiation of treatment-related changes from glioma progression

**DOI:** 10.1007/s11060-022-04089-2

**Published:** 2022-07-19

**Authors:** Marguerite Müller, Oliver Winz, Robin Gutsche, Ralph T. H. Leijenaar, Martin Kocher, Christoph Lerche, Christian P. Filss, Gabriele Stoffels, Eike Steidl, Elke Hattingen, Joachim P. Steinbach, Gabriele D. Maurer, Alexander Heinzel, Norbert Galldiks, Felix M. Mottaghy, Karl-Josef Langen, Philipp Lohmann

**Affiliations:** 1 Department of Nuclear Medicine and Comprehensive Diagnostic Center Aachen (CDCA), RWTH Aachen University, Aachen, Germany; 2 Center for Integrated Oncology (CIO), Universities of Aachen, Bonn, Cologne, and Duesseldorf, Germany; 3 Institute of Neuroscience and Medicine (INM-3, -4, -11), Research Center Juelich (FZJ), Juelich, Germany; 4 RWTH Aachen University, Aachen, Germany; 5 Department of Radiation Oncology (MAASTRO), Maastricht University Medical Center (MUMC+), Maastricht, The Netherlands; 6 Department of Stereotaxy and Functional Neurosurgery, Faculty of Medicine and University Hospital Cologne, University of Cologne, Cologne, Germany; 7 Institute of Neuroradiology, University Hospital, Goethe University Frankfurt am Main, Frankfurt am Main, Germany; 8 University Cancer Center Frankfurt (UCT), University Hospital, Goethe University Frankfurt am Main, Frankfurt am Main, Germany; 9 German Cancer Consortium (DKTK), Partner Site Frankfurt/Mainz, German Cancer Research Center (DKFZ), Heidelberg, Germany; 10 Dr. Senckenberg Institute of Neurooncology, University Hospital, Goethe University Frankfurt am Main, Frankfurt am Main, Germany; 11 Department of Neurology, Faculty of Medicine and University Hospital Cologne, University of Cologne, Cologne, Germany; 12 Department of Radiology and Nuclear Medicine, Maastricht University Medical Center (MUMC+), Maastricht, The Netherlands

**Keywords:** Amino acid PET, Brain tumors, Artificial intelligence (AI), Machine learning

## Abstract

**Purpose:**

To investigate the potential of radiomics applied to static clinical PET data using the tracer O-(2-[^18^F]fluoroethyl)-l-tyrosine (FET) to differentiate treatment-related changes (TRC) from tumor progression (TP) in patients with gliomas.

**Patients and Methods:**

One hundred fifty-one (151) patients with histologically confirmed gliomas and post-therapeutic progressive MRI findings according to the response assessment in neuro-oncology criteria underwent a dynamic amino acid PET scan using the tracer O-(2-[^18^F]fluoroethyl)-l-tyrosine (FET). Thereof, 124 patients were investigated on a stand-alone PET scanner (data used for model development and validation), and 27 patients on a hybrid PET/MRI scanner (data used for model testing). Mean and maximum tumor to brain ratios (TBR_mean_, TBR_max_) were calculated using the PET data from 20 to 40 min after tracer injection. Logistic regression models were evaluated for the FET PET parameters TBR_mean_, TBR_max_, and for radiomics features of the tumor areas as well as combinations thereof to differentiate between TP and TRC. The best performing models in the validation dataset were finally applied to the test dataset. The diagnostic performance was assessed by receiver operating characteristic analysis.

**Results:**

Thirty-seven patients (25%) were diagnosed with TRC, and 114 (75%) with TP. The logistic regression model comprising the conventional FET PET parameters TBR_mean_ and TBR_max_ resulted in an AUC of 0.78 in both the validation (sensitivity, 64%; specificity, 80%) and the test dataset (sensitivity, 64%; specificity, 80%). The model combining the conventional FET PET parameters and two radiomics features yielded the best diagnostic performance in the validation dataset (AUC, 0.92; sensitivity, 91%; specificity, 80%) and demonstrated its generalizability in the independent test dataset (AUC, 0.85; sensitivity, 81%; specificity, 70%).

**Conclusion:**

The developed radiomics classifier allows the differentiation between TRC and TP in pretreated gliomas based on routinely acquired static FET PET scans with a high diagnostic accuracy.

## Introduction

During the follow-up of glioma patients, treatment-related changes often cannot be reliably differentiated from tumor progression by structural magnetic resonance imaging (MRI) alone. Yet a false diagnosis will either result in the continuation of an ineffective treatment or a premature termination of an effective one, both negatively impacting patients’ prognosis. To improve the differentiation of treatment-related changes and tumor progression, advanced MRI techniques such as perfusion-weighted imaging as well as MR spectroscopy are under investigation, yielding accuracies of about 80% for this clinically challenging and highly important differential diagnosis [1].

Besides advanced MRI, amino acid positron emission tomography (PET) has demonstrated its potential to differentiate treatment-related changes from tumor progression in initial studies [2–4]. Previous studies using the amino acid PET tracer O-(2-[^18^F]fluoroethyl)-l-tyrosine (FET) showed that the combination of static and dynamic parameters discriminates treatment-related changes from tumor progression in recurrent gliomas with an accuracy of up to 90% [5–7]. These studies, however, require dynamic FET PET parameters based on a 40–50 min PET scan that is more time consuming in clinical routine than a static scan from 20 to 40 min post injection. Other studies demonstrated an improved diagnostic accuracy by combination of FET PET with advanced MRI methods using hybrid PET/MRI [8, 9].

Despite these recent advances, a method to improve the diagnostic performance of FET PET without the need for a time-consuming and expensive dynamic acquisition or additional, dedicated MRI scans would be of clinical relevance.

In recent years, methods based on artificial intelligence and machine learning have become increasingly important and found their way into medical image analysis. Several methods from this field are under investigation also in brain tumor patients and promise to improve diagnosis by extracting additional imaging features from routinely acquired imaging data. These features are usually not accessible through conventional image analysis and can be used to generate prognostic or predictive mathematical models. This methodology is also referred to as radiomics [10, 11].

FET PET radiomics has already demonstrated its potential in neurooncology for the prediction of the isocitrate dehydrogenase genotype [12], the diagnosis of pseudoprogression [13, 14], the differentiation of treatment-related changes from recurrent brain metastases after radiosurgery [15, 16], or the prediction of the BRAF mutational status in patients with melanoma brain metastases [17].

The goal of our study was to investigate the potential of FET PET radiomics for the differentiation between treatment-related changes and tumor progression in patients with glioma based on routinely acquired static FET PET when added to clinically established FET PET parameters.

## Patients and methods

### Patients

The patient group was partly included in a previous study concerning the diagnostic performance of perfusion-weighted MRI and dynamic FET PET for the differentiation of treatment-related changes from glioma progression [18].

One hundred and fifty-one patients (*n* = 54 females, *n* = 97 males; median age, 52.3 years; age range 20.4–78.0 years) with histologically confirmed WHO grade II–IV glioma according to the 2016 WHO Classification of Tumors of the Central Nervous System [19] were included in this retrospective study [mostly WHO grade IV glioblastoma, IDH-wildtype (n = 71); WHO grade III anaplastic astrocytoma, IDH-mutant (n = 20); WHO grade II astrocytoma, IDH-mutant (n = 14)]. All patients presented with post-therapeutic MRI findings suspicious for tumor progression according to the Response Assessment in Neuro-Oncology (RANO) criteria and were hence investigated using FET PET. All patients included here showed an increased FET uptake in the area of the primary lesion. Complete patient characteristics are provided in Table 1.

**Table 1:** Patient characteristics

Demographics	
Number of patients	151
Sex (female/male)	54/97
Age (years) (median and range)	52.3 (20.4–78.0)
Histology	
Oligodendroglioma, IDH-mutant and 1p/19q-codeleted	
WHO grade II	7 (5%)
WHO grade III	10 (7%)
Astrocytoma IDH-mutant	
WHO grade II	14 (9%)
WHO grade III	20 (13%)
Astrocytoma IDH-wildtype	
WHO grade II	5 (3%)
WHO grade III	10 (7%)
Astrocytoma, NOS, WHO grade II	2 (1%)
Glioblastoma, IDH-wildtype, WHO grade IV	71 (47%)
Glioblastoma, IDH-mutant, WHO grade IV	11 (7%)
Gliosarcoma, WHO grade IV	1 (1%)
Molecular characteristics	
IDH genotype	
IDH-mutant	59 (39%)
IDH-wildtype	92 (61%)
MGMT promoter methylation status	
Methylated	72 (48%)
Unmethylated	50 (33%)
Not available	29 (19%)
Final diagnosis	
Tumor progression	114 (75%)
Treatment-related changes	37 (25%)
Diagnosis based on histopathology	46 (30%)
Diagnosis based on clinicoradiological follow-up	105 (70%)

### Diagnosis of treatment-related changes and tumor progression

Diagnosis was based on histopathology in 46 patients (30%), and on clinicoradiological follow-up in 105 patients (70%). For histopathologic diagnosis, tissue samples were obtained by resection or biopsy and analyzed as described previously [5].

For WHO grade II gliomas, both the clinical and the radiological situation had to be stable or improved for at least 12 months without change in therapy to exclude tumor progression [20]. For WHO grade III–IV gliomas, the diagnosis treatment-related changes required at least 6 months of stable or improved clinical and radiological condition [21], as well as no change in tumor treatment. Tumor progression was diagnosed if lesions continued to increase in size on at least two subsequent MRI scans according to the RANO criteria, accompanied by a deterioration in performance status, or if a patient died of glioma, whichever occurred first. Of note, the applied classification criteria are in accordance with previous studies [5, 22, 23].

### FET PET imaging

The amino acid FET was produced and applied as described previously [24]. According to international guidelines for brain tumor imaging, all patients fasted for at least 4 h before the PET measurement [25].

All patients underwent a dynamic PET scan from 0 to 50 min post injection of 3 MBq of FET per kg of body weight. 124 patients were examined on a stand-alone PET scanner (ECAT EXACT HR+, Siemens Healthcare, Erlangen, Germany) in 3D mode, and 27 patients on a high-resolution 3 T hybrid PET/MRI scanner (BrainPET, Siemens Healthcare, Erlangen, Germany). The BrainPET is a compact cylinder that fits into the bore of the Magnetom Trio MR scanner [26, 27].

As described before [28], iterative reconstruction parameters were: 16 subsets, six iterations using the OSEM algorithm for the ECAT HR+ PET scanner and two subsets, and 32 iterations using the OP-OSEM algorithm for the BrainPET. Data were corrected for random, scattered coincidences, dead time, and motion for both systems. Attenuation correction for the ECAT HR+ PET was based on a transmission scan. For the BrainPET, a template-based approach was used [26]. The reconstructed dynamic data sets consisted of 16 time frames (5 × 1 min; 5 × 3 min; 6 × 5 min) for both scanners. To optimize comparability of the results related to the influence of the two different PET scanners, reconstruction parameters, and post-processing steps, a 2.5 mm 3D Gaussian filter was applied to the BrainPET data before further processing. In previous phantom experiments with spheres of different sizes that simulated lesions, this filter kernel demonstrated the best comparability of PET data obtained from the ECAT HR+ PET and the BrainPET scanner [29].

### Evaluation of FET PET parameters

The FET uptake was expressed as standardized uptake value (SUV) by dividing the radioactivity in the tissue (kBq/ml) by the radioactivity injected per gram of body weight.

Semi-automated segmentation of the suspected brain lesion was performed in the summed PET images from 20 to 40 min post-injection using the Pmod Biomedical Image Quantification Software (Version 3.806, PMOD Technologies, Zurich, Switzerland). For assessment of the FET uptake in healthy brain tissue, a region-of-interest was positioned in the semioval center of the unaffected hemisphere contralateral to the tumor, involving grey and white matter [25]. As described before [28], tumor segmentation was performed using a 2-dimensional auto-contouring process in the transversal slice containing the voxel with the maximum tracer uptake using a tumor-to-brain ratio (TBR) of 1.6 or more. In a previous study, this threshold has been shown to best separate between vital tumor and healthy brain parenchyma in FET PET [30].

**Fig. 1:**
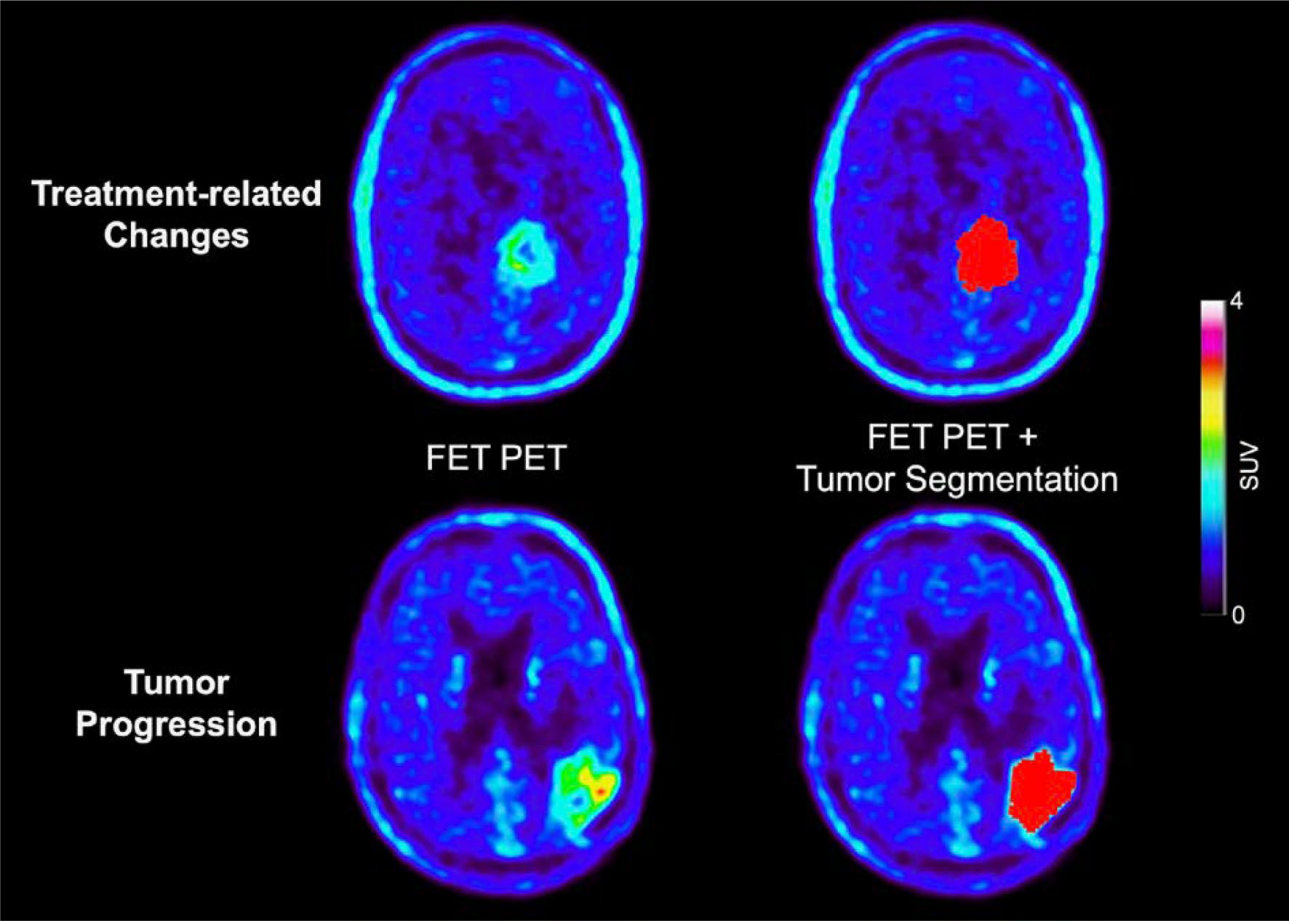
Representative FET PET images of patients with treatment-related changes (top) and glioma progression (bottom). The segmented lesions are highlighted in red in the right column. Visually, obvious differences in FET uptake between patients with treatment-related changes and tumor progression could not be identified

Maximum and mean TBRs (TBR_max_, TBR_mean_) were calculated by dividing the maximum or mean SUV of the tumor by the mean SUV of healthy brain tissue. The segmentations were validated by an experienced, board-certified specialist in neuroradiology with broad experience in nuclear medicine. Figure 1 illustrates the segmented lesions in representative patients.

### Image preprocessing and radiomics feature extraction

The group of patients scanned on the stand-alone PET scanner was divided into a training and a validation dataset in a ratio of 3/1 with an equal ratio of tumor progression to treatment-related changes. The group of patients scanned on the 3 T hybrid PET/MR scanner was used for model testing.

Feature extraction was performed by the RadiomiX toolbox (supported by Oncoradiomics, Liège, Belgium) [31] implemented in Matlab 2017a (MathWorks, Natick, MA, USA), including International Biomarker Standardization Initiative (IBSI)-compliant [32] radiomic features as well as others. A total of 221 features were extracted. No spatial resampling was performed. Absolute intensity resampling was performed using a fixed bin width of 0.1 according to current recommendations [33].

The extracted features consisted of five main groups: (1) fractal features (2) first order statistics, (3) shape and size, (4) texture descriptors including gray level co-occurrence (GLCM), gray level run-length (GLRLM), and gray level size-zone texture matrices (GLSZM), and (5) features from groups 1, 3 and 4 after wavelet decomposition of the original image.

The definitions and detailed feature descriptions are provided elsewhere [10]. Detailed mathematical definitions of the features are available in the RadiomiX toolbox manual.

### Feature selection

Using large number of features on a limited number of patients for model calculation may result in data overfitting. Overfitting is a methodological mistake in which a generated model corresponds too closely or even matches the analyzed dataset. This results in a perfect classification accuracy on the dataset that has been used for training but renders the model too specialized to classify new or additional imaging data or reliably predict future observations. To lower the risk of overfitting, the most important features must be identified in a process called feature selection before model generation. [34]

Feature selection was performed using the RadiomiX toolbox. First, the machine learning module eliminates features with (near) zero variance and an inter-feature correlation of 0.9 or more to remove redundancies within the feature set. A threshold of 0.9 is generally considered high enough to eliminate high correlation from the dataset. Second, a stepwise forward feature selection using stratified cross-validation with logistic regression is used to further reduce the number of radiomics features, i.e., it stops adding features if the inclusion of the next feature does not add more than 0.005 to the average cross-validation AUC.

### Model generation and validation

Logistic regression models were generated on the training dataset using the ‘tidyverse/ggplot2’ packages in R (version 4.0.5, R Studio, Inc., Boston, MA, USA). Logistic regression models were fitted separately for the conventional FET PET parameters TBR_mean_ and TBR_max_, as well as for the selected radiomics features and a combination of conventional and radiomics features. Finally, the models were applied to the holdout validation dataset that was not part of model generation.

### Model testing

**Fig. 2:**
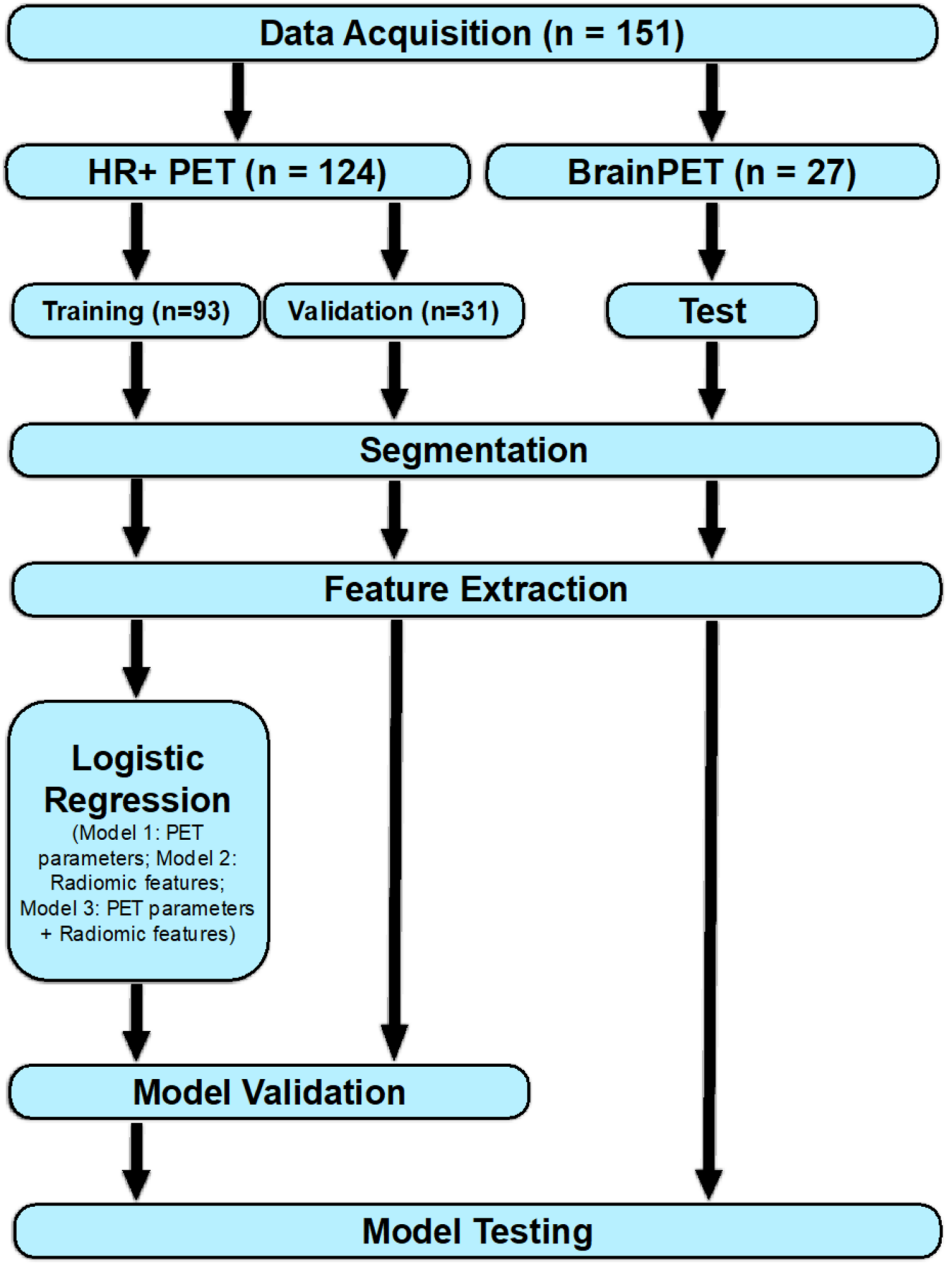
Radiomics workflow

The best performing models were applied to the test dataset acquired on the BrainPET scanner. Since the test dataset was not involved in the process of model training and validation and was acquired on a different PET scanner, it represents an independent dataset to evaluate the robustness and generalizability of the model. The radiomics workflow is illustrated in Fig. 2.

### Statistical evaluation

Descriptive statistics are provided as mean and standard deviation or as median and range. The Mann–Whitney-U test was used for intergroup comparison. The diagnostic performance of the PET parameters, the machine learning models and combinations thereof were assessed by receiver operating characteristic (ROC) analysis. The decision cutoff was considered optimal when the product of paired values for sensitivity and specificity reached its maximum. Fisher’s exact test for 2 × 2 contingency tables was used for statistical evaluation of the parameters. *P*-values of less than 0.05 were considered statistically significant. Statistical analyses were performed using SPSS (SPSS Statistics 24, IBM, New York, USA) and Microsoft Excel (Excel:Mac 2020, Version 16.53, Microsoft, Redmond, WA, USA).

## Results

### Treatment-related changes and tumor progression

Of the 124 patients examined on the stand-alone PET scanner, 31 (25%) were diagnosed with treatment-related changes and 93 (75%) with tumor progression. The test dataset consisted of 27 patients examined on the BrainPET scanner. Thereof, six (22%) were diagnosed with treatment-related changes and 21 (78%) with tumor progression.

### Group comparison of FET PET parameters

TBR_mean_ was significantly higher for patients with tumor progression compared to patients with treatment-related changes (mean TBR_mean_ ± standard deviation, 2.1 ± 0.3 vs. 1.9 ± 0.3; p < 0.001). TBR_max_ was also significantly higher for patients with tumor progression compared to patients with treatment-related changes (mean TBR_max_ ± standard deviation, 3.7 ± 0.9 vs. 2.8 ± 0.7; p < 0.001).

### Performance of machine learning models in the training and validation dataset

The two most important radiomics features according to the feature selection were *Informational Measure of Correlation 2* calculated from the GLCM, and *Intensity Non-Uniformity Normalized* from the GLSZM.

**Fig. 3:**
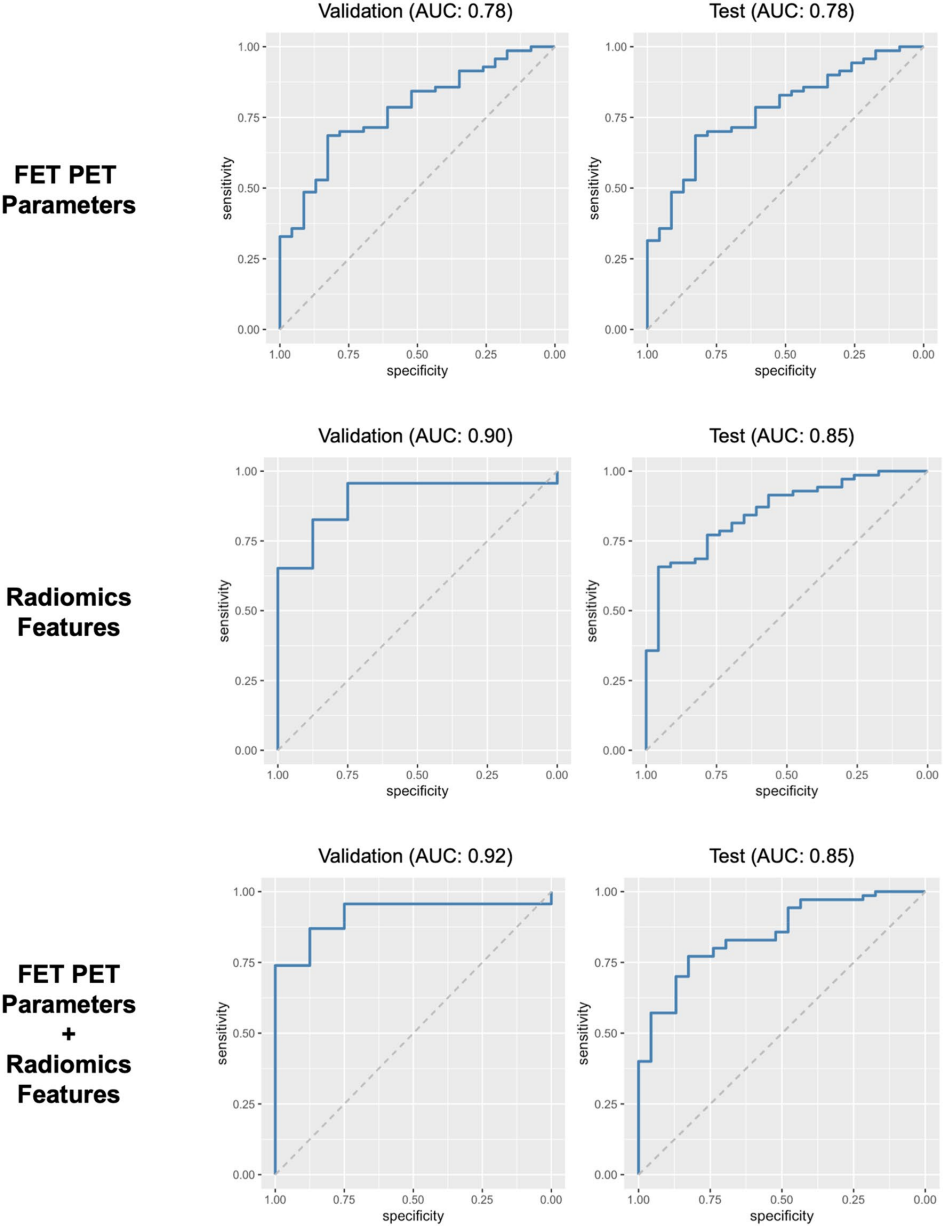
Receiver operating characteristic curves of the validation (left) and the test dataset (right) for a model comprising the static FET PET parameters TBR_mean_ and TBR_max_ (top row), a model comprising the two radiomics features *Informational Measure of Correlation 2* calculated from the grey level co-occurrence matrix, and *Intensity Non-Uniformity Normalized* from the grey level size zone matrix (middle row), and a model using the combination thereof (bottom row)

The logistic regression model based on the conventional PET parameters resulted in an AUC of 0.78 (95% confidence interval, 0.68–0.88; sensitivity, 64%; specificity, 80%) in the validation dataset. The logistic regression model using only radiomics features resulted in an AUC of 0.90 (95% confidence interval, 0.79–1.00; sensitivity, 87%; specificity, 80%) in the validation dataset. The logistic regression model combining FET PET parameters and radiomics features resulted in an AUC of 0.92 (95% confidence interval, 0.82–1.00; sensitivity, 91%; specificity, 80%) in the validation dataset. Further details on the model performances in the validation and test dataset are summarized in Table 2 and Fig. 3.

**Table 2:** Diagnostic performance of developed classifiers in the validation dataset (top) and the test dataset (bottom)

	AUC	95% CI	Sensitivity (%)	Specificity (%)
Validation dataset (n = 31)				
FET PET parameters	0.78	0.68–0.88	64	80
Radiomics features	0.90	0.79–1.00	87	80
FET PET parameters + radiomics features	0.92	0.82–1.00	91	80
Test dataset (n = 27)				
FET PET parameters	0.78	0.67–0.88	66	80
Radiomics features	0.85	0.77–0.94	73	80
FET PET parameters + radiomics features	0.85	0.77–0.94	81	70

*AUC*: area under the receiver operating characteristic curve; *95% CI*: 95% confidence interval

### Performance of radiomics models in the test dataset

The model based on PET parameters resulted in an AUC of 0.78 (95% confidence interval, 0.67–0.88; sensitivity, 66%; specificity, 80%) in the test dataset.

The model based solely on radiomics features resulted in an AUC of 0.85 (95% confidence interval, 0.77–0.94; sensitivity, 73%; specificity, 80%) in the test dataset. The model combining FET PET parameters and radiomics features resulted in an AUC of 0.85 (95% confidence interval, 0.77–0.94; sensitivity, 81%; specificity, 70%). Further details on the model performances in the test dataset are summarized in Table 2 and Fig. 3.

## Discussion

The main finding of our study is that a machine learning model based on static FET PET radiomics features differentiates treatment-related changes from tumor progression in patients with gliomas with a high diagnostic accuracy, i.e., an AUC of 0.85, and outperforms conventional FET PET analysis (AUC, 0.78). Interestingly, combining both FET PET radiomics features with conventional FET PET parameters further improved the overall diagnostic performance, especially the sensitivity of the model. Further, the developed radiomics model was evaluated in a small, but independent test dataset acquired with a different PET scanner demonstrating its reliability and robustness regardless of the used scanners and imaging parameters. Since the developed model is based on routinely acquired FET PET scans and can be applied fully automated on a conventional computer in a few minutes, the approach seems feasible for clinical implementation.

Several studies already investigated the potential of FET PET for the differentiation of treatment-related changes from tumor progression in glioma patients [7, 20, 35, 36]. Although these studies demonstrate high diagnostic accuracies, the number of patients in these studies was either low, and/or only a small fraction of patients were diagnosed with treatment-related changes. A more recent study by Maurer and colleagues [5] using partially overlapping patients investigated the value of static and dynamic FET PET parameters for the differentiation of treatment-related changes from glioma progression. This retrospective analysis of 127 patients with WHO grade II-IV gliomas yielded a diagnostic accuracy of 81% (sensitivity, 86%; specificity, 67%) by combining TBR_max_ and the dynamic FET PET parameter slope. In our study, the combination of static FET PET parameters TBR_mean_ and TBR_max_ resulted in a comparable diagnostic performance (AUC, 0.78; sensitivity, 64%; specificity, 80%) in the validation dataset. Nevertheless, the use of FET PET radiomics parameters alone already outperformed the conventional FET PET parameters with an AUC of 0.90 (sensitivity, 87%; specificity, 80%). Combining radiomics and conventional FET PET parameters further increased the diagnostic performance in the validation dataset (AUC, 0.92; sensitivity, 91%; specificity, 80%) and demonstrated its generalizability in the external test dataset (AUC, 0.85; sensitivity, 81%; specificity, 70%).

Our results concerning FET PET are also comparable with a recent study of our group investigating the value of combining perfusion-weighted MRI with dynamic FET PET [37]. In that study, accuracy of PWI, which can be performed easily during routine conventional MR scanning, was poor in differentiating treatment-related changes and tumor progression (accuracy, 63%). However, the high positive predictive value of PWI (100%) allowed a correct diagnosis of treatment-related changes in 42% of the patients. In the remaining patients, PWI was nondiagnostic, but FET PET still achieved an accuracy of 78% leading to the recommendation of a sequential use of perfusion-weighted MRI and dynamic FET PET in clinical practice. In this context, the developed radiomics classifier in our study may achieve a higher diagnostic performance based on 20 min static FET PET images. Since the analysis can be performed fully automated on a conventional computer in a few minutes, this combination appears promising in terms of a clinical translation.

Over the past years, the value of machine learning techniques and FET PET radiomics for the diagnosis of treatment-related changes such as pseudoprogression in patients with glioma [13, 14, 38] or radiation injury in patients with brain metastases [16] has been demonstrated. Interestingly, in these studies, different patterns of tracer uptake could already visually be distinguished. Patients with pseudoprogression or radiation injuries showed a more homogenous uptake of FET compared to a more heterogenous uptake of FET in patients with tumor progression.

In our study, different patterns of FET uptake between patients with treatment-related changes and glioma progression could not be identified by visual evaluation (Fig. 1). This might be due to the more inhomogeneous group of patients that included a broad range of glioma subtypes and treatment regimens. Nevertheless, we identified the two textural features *Informational Measure of Correlation 2* calculated from the GLCM, and *Intensity Non-Uniformity Normalized* from the GLSZM as being discriminative between treatment-related changes and glioma progression. Both textural features describe differences in tumor heterogeneity not accessible by means of human perception. Even though visually accessible differences are desirable for a better presentation of the results, the real benefit and concept of radiomics becomes more apparent if this is not the case—”images are more than pictures, they are data” [39].

Besides a visual interpretation of radiomics features, further efforts are needed for a deeper understanding of the biological meaning of features and machine learning models. This might be essential for a potential clinical translation and acceptance of radiomics in clinical routine. Hence, the correlation of radiomics features with local tissue samples including extensive neuropathological work-up is necessary in future studies.

Although promising, our results must be further validated in a larger group of patients from multiple institutions. Albeit the number of patients in our study is larger than in other studies investigating PET radiomics for the differentiation of treatment-related changes and tumor progression, the generally low number of patients available in neuro-oncology remains a limitation. Nonetheless, our model has shown its value in an external test dataset without extensive preprocessing, acquired on a different PET scanner, so further model evaluation in other centers is warranted and feasible.

Another limitation of our study might be the heterogenous composition of patients in terms of glioma subtypes and treatment regimens and the relatively low amount of histopathological validation of the diagnosis. Further, the group of patients is likely biased towards more challenging cases as only patients with equivocal MRI findings and remaining therapeutic options usually undergo FET PET scans. However, this dataset is representative of a clinical situation and further underlines the value of the model as it does not require an extensive preselection of patients.

Additionally, the developed machine learning model is based on FET PET alone and does not include structural or advanced MRI. Future studies should hence address the potential additional value of a combined FET PET/MRI radiomics analysis, also considering advanced MRI methods such as PWI or MR spectroscopy.

## Conclusion

The results from our study suggest that the developed radiomics model is of clinical value for the differentiation between treatment-related changes and tumor progression in patients with gliomas regardless of tumor type or pretreatment. The radiomics model is based on routinely acquired static 20 min FET PET scans facilitating the translation into clinical routine. Especially in combination with other clinical parameters, the developed radiomics model might have an additional diagnostic value once translated into clinical routine.

## Data Availability

All data supporting the findings presented in this manuscript are available upon request directly to the corresponding author, PL. These data are not part of public domain or database as they are part of the patient protected medical record and public sharing would compromise the privacy of the research participants.
